# Targeting Oncogenic lncRNA KRT7-AS to Induce Ferroptosis Suppresses Ovarian Cancer Progression

**DOI:** 10.32604/or.2026.075241

**Published:** 2026-04-22

**Authors:** Yan Zhu, Bin Guan, Wencai Guan, Jihong Zhang, Shiyu Wang, Jimin Shi, Wei Fan, Qi Lu, Lingyun Zhang, Guoxiong Xu

**Affiliations:** 1 Research Center for Clinical Medicine, Jinshan Hospital, Fudan University, Shanghai, China; 2 Department of Oncology, Shanghai Medical College, Fudan University, Shanghai, China; 3 Department of Obstetrics and Gynecology, Jinshan Hospital, Fudan University, Shanghai, China; 4 Department of Medical Oncology, Shanghai Geriatric Medical Center, Shanghai, China; 5 Department of Medical Oncology, Zhongshan Hospital, Fudan University, Shanghai, China

**Keywords:** Biomarker, ferroptosis, non-coding RNA, oncogenesis, tumor suppression

## Abstract

**Background:**

Ovarian cancer poses the greatest threat to survival among gynecologic cancers in women. Long non-coding RNAs (lncRNAs) have emerged as critical regulators in oncogenesis. The current study aimed to elucidate the function and regulatory mechanism of lncRNA KRT7-AS in ovarian cancer.

**Methods:**

The clinical significance of KRT7-AS was evaluated through bioinformatics analysis of data from public repositories. KRT7-AS expression was examined by RT-qPCR and fluorescence *in situ* hybridization. The function analyses were conducted using assays for cell proliferation, migration, invasion, wound healing, and colony formation. Assessment of cell cycle and apoptosis was performed using flow cytometry. Mitochondrial membrane potential (MMP), reactive oxygen species (ROS), lipid peroxidation, and ferrous iron (Fe^2+^) levels were measured with specific kits. Tumor growth was assessed using a xenograft mouse model.

**Results:**

Patients exhibiting high KRT7-AS expression had a significantly lower survival rate. Functional assays demonstrated that KRT7-AS overexpression enhanced tumorigenic behaviors, including cell proliferation, invasion, and metastasis, whereas its knockdown suppressed these malignant phenotypes. KRT7-AS depletion induced ferroptosis, as indicated by increased MMP and ROS levels, and the accumulation of lipid peroxidation and Fe^2+^. In rescue experiments, the ferroptosis inhibitor ferrostatin-1 reversed the reduction in cell viability caused by KRT7-AS knockdown. Finally, *in vivo* studies showed that KRT7-AS knockdown inhibited tumor growth and modulated the expression of ferroptosis-related proteins by elevating ACSL4 and reducing GPX4.

**Conclusions:**

These findings suggest that KRT7-AS has potential as a diagnostic biomarker for ovarian cancer. Targeting KRT7-AS to induce ferroptosis may represent a promising therapeutic strategy for suppressing ovarian cancer progression.

## 1 Introduction

Ovarian cancer, a heterogeneous gynecologic malignancy, is the most lethal disease in women with unfavorable prognoses [1]. With approximately 324,398 new cases diagnosed each year globally in 2022, over 70% of patients have the disease at the advanced stage initially [2]. Current treatment involves cytoreductive surgery followed by platinum-based chemotherapy or interval debulking after neoadjuvant chemotherapy. However, approximately 80% of patients experience recurrence and subsequently develop chemoresistance, persistently driving the 5-year survival rate below 40% [3]. This clinical reality highlights the urgent need for molecular understanding of ovarian tumorigenesis to guide the development of novel therapeutic strategies and precision medicine approaches by eliminating cancer cells [4,5].

Cell death manifests through two distinct types, accidental cell death (ACD) and regulated cell death (RCD) [6,7]. ACD results from exposure to extreme physicochemical insults, whereas RCD involves genetically encoded molecular programs that regulate tissue homeostasis and contribute to pathogenic processes. The RCD comprises apoptotic and non-apoptotic subtypes, exhibiting distinct signaling initiators, molecular regulators, and disease associations [8]. Notably, non-apoptotic RCD modalities, including ferroptosis, pyroptosis, and necroptosis, play critical roles in oncogenesis and tumor progression [9]. Ferroptosis, an iron-catalyzed form of cell death, displays necrotic morphological features such as cellular swelling and plasma membrane rupture, driven by lethal accumulation of lipid peroxidation [10]. This oxidative death mechanism has been increasingly recognized as a crucial determinant in cancer pathogenesis and therapeutic responses [11]. This process involves multiple molecular regulators. For instance, glutathione peroxidase 4 (GPX4) is a negative regulator that suppresses ferroptosis by reducing reactive oxygen species (ROS) levels [12], whereas acyl-CoA synthetase long-chain family member 4 (ACSL4) is a positive factor that catalyzes long-chain fatty acids to produce lipid peroxides to induce ferroptosis [13]. Recent studies have shown that dysregulated non-coding RNAs, particularly long non-coding RNAs (lncRNAs), play an important role in modulating cell death pathways, including ferroptosis, in the initiation and progression of multiple cancers [14,15].

LncRNAs have emerged as critical regulators in oncogenesis by modulating cellular processes such as proliferation, migratory capacity, and genomic integrity [16]. Recent advances in high-throughput transcriptomic analyses have systematically identified thousands of dysregulated lncRNAs across various malignancies [17,18]. The oncogenic mechanisms of lncRNAs encompass three primary pathways: (1) epigenetic regulation through chromatin modifications and DNA methylation patterns; (2) stabilization of protein complexes via molecular scaffolding; and (3) competitive endogenous RNA (ceRNA) activity that sequesters tumor-suppressive microRNAs (miRNAs). Previous studies showed that lncRNA keratin-7 antisense (KRT7-AS) has been partly characterized in gastric, lung, and breast cancers [19,20]. However, the role of KRT7-AS in ovarian cancer remains unexplored.

This study, therefore, aims to define KRT7-AS as a biomarker and elucidate its regulatory mechanism, specifically through ferroptosis, in ovarian cancer.

## 2 Material and Methods

### 2.1 RNA-Seq Data Acquisition

Data acquisition of RNA-sequencing expression and corresponding clinical details on ovarian cancer were from the TCGA database (https://tcga-data.nci.nih.gov/tcga/) and were analyzed using the R package of “GSVA” with the parameter method = “ssgsea” (Version 1.46.0, https://github.com/rcastelo/GSVA), which was performed using Hallmark gene sets from the Molecular Signatures Database (MSigDB) (http://www.gsea-msigdb.org/gsea/msigdb). All data of transcriptomic profiles from TCGA (ovarian cancer) and GTEx (normal ovarian tissue) were downloaded from UCSC Xena (https://xena.ucsc.edu). The cohort comprised 374 cancer samples and 88 normal samples after excluding cases with incomplete survival annotations. To analyze the prognosis correlated with lncRNAs between high and low-expression groups, a median cutoff value was applied. To functionally contrast the TCGA and GTEx datasets, we focused on signatures critically involved in ovarian cancer pathogenesis, including EPITHELIAL_MESENCHYMAL_TRANSITION, DNA_REPAIR, and APOPTOSIS. Heatmap analysis of GTEx and TCGA datasets identified differential lncRNA expression between normal and OC tissues using log_2_-transformed normalized counts by log_2_(TPM + 1) with a threshold of absolute value of fold change greater than 2 (|Fold Change| > 2, *p* < 0.001). Differentially expressed genes were then identified using DESeq2 (Version 1.38.3), with a significance threshold set at a False Discovery Rate (FDR)-adjusted *p*-value (*q*-value) < 0.05. The “ggrisk”, “survival”, and “survminer” R packages (Version 4.2.3; https://cran.r-project.org/web/packages/survminer/index.html) were employed to analyze the difference in survival rate between two groups using the log-rank test.

### 2.2 Cell Lines and Cell Culture

IOSE-80, a normal ovarian surface epithelial cell line, was purchased (Cat# FH1132; RRID: CVCL-5546, FuHeng BioLogy, Shanghai Fuheng Biology Science and Technology Co., Ltd., Shanghai, China) and cultured in Roswell Park Memorial Institute 1640 medium (RPMI-1640; Cat# KGL1503-500, KeyGen Biotech., Ltd., Nanjing, China). OVCAR-3 cell line (Cat# SCSP-558; RRID: CVCL-0465; National Collection of Authenticated Cell Cultures, Shanghai, China) originated from a patient with high-grade serous ovarian adenocarcinoma and was cultured in RPMI-1640. A2780 cell line (Cat# FH0140; RRID: CVCL-0134, FuHeng BioLogy) originated from a patient with ovarian endometrioid adenocarcinoma and was cultured in Dulbecco’s Modified Eagle’s Medium (DMEM; Cat# KGL1211-500, KeyGen Biotech). The human-derived cell lines were obtained commercially where relevant ethical compliance was obtained by the providing institution and used in accordance with the principles of the Declaration of Helsinki. All media supplemented with 10% fetal bovine serum (FBS; Cat# F8318, Invitrogen, Carlsbad, CA, USA). Each cell line was authenticated via short tandem repeat (STR) profiling and routinely tested to ensure mycoplasma-free status.

### 2.3 Transfection and Infection

Small-interfering RNAs (siRNAs) of KRT7-AS (si-KRT7-AS) and negative control (si-NC) were produced by Shanghai GenePharma Co., Ltd. (Shanghai, China). Three specific siRNA sequences are listed in Table A1. Preliminary transfection efficiency validation identified the si-KRT7-AS-3 sequence as the most effective after screening; this sequence was subsequently used to construct lentiviral-packaged short-hairpin RNA (shRNA) of KRT7-AS (sh-KRT7-AS). The sequence of sh-KRT7-AS is listed in Table A2. The KRT7-AS overexpression plasmid (oe-KRT7-AS) was constructed by cloning the full-length transcript into the pcDNA3.1 vector (Cat# 80-466596868, Hanyin Biotechnology, Shanghai, China) with forward and reverse primers (Table A3). OVCAR-3 cells (5 × 10^4^/well) and A2780 cells (3 × 10^4^/well) in a 6-well plate were transfected for 24 h. Transient transfections employed X-tremeGENE reagent (Cat# 04476093001, Roche Applied Science, Indianapolis, IN, USA) for siRNA and Lipo8000 reagent (Cat# C0533, Beyotime Biotechnology, Shanghai, China) for plasmid according to the manufacturer’s instructions.

### 2.4 Reverse Transcript-Quantitative Polymerase Chain Reaction (RT-qPCR)

Total RNA extraction procedure was carried out utilizing the RNA-Quick Purification Kit (Cat# RT001, Yishan Biotechnology Co., Ltd., Shanghai, China) according to the manufacturer’s protocol. The quality of RNA was assessed by measuring the absorbance values of 260 nm and 280 nm with the reference values for an OD260/OD280 ratio between 1.8 and 2.0. Subsequent complementary DNA (cDNA) synthesis was performed using 10 ng DNA per reaction with the First Strand cDNA Synthesis Kit (Cat# 04896866001, Roche, Mannheim, Germany) according to the manufacturer’s instructions. Nucleus and cytoplasmic RNAs were fractionated using a Cytoplasmic & Nuclear RNA Purification Kit (Cat# 21000, Norgen Biotek Corp., Thorold, ON, Canada). PCR condition was as follows: initial denaturation at 95°C for 2 min and 40 cycles of amplification with denaturation at 95°C for 15 s and annealing/extension at 60°C for 30 s. Gene expression levels were quantified by real-time PCR on a QuantStudio 3 System (Applied Biosystems, Waltham, MA, USA) using BeyoFast™ SYBR Green qPCR Mix (Cat# D7262, Beyotime Biotechnology). Actin served as the endogenous control. Primer sequences are detailed in Table A4.

### 2.5 Protein Extraction and Western Blotting

Cell lysis was performed with ice-cold SDS Lysis Buffer (1 × 10^6^/200 μL) (Cat# P0013G, Beyotime Biotechnology) containing a protease/phosphatase inhibitor cocktail. Protein concentration was measured using a BCA kit (Cat# P0010; Beyotime Biotechnology). Proteins (30 μg/lane) were run on 4%–20% precast gels and transferred to polyvinylidene fluoride (PVDF; Cat# IPVH00010, Millipore, Burlington, MA, USA) membranes. Subsequently, the membrane was blocked in QuickBlock™ buffer (Cat# P0252-500mL, Beyotime Biotechnology) for 1 h at room temperature and incubated with specific primary antibody at 4°C overnight. The primary antibodies used were: anti-ACSL4 (dilution 1:2000; Cat# 22401-1-AP, Proteintech, Wuhan, China), anti-GPX4 (dilution 1:1000; Cat#67763-1-lg, Proteintech), or anti-Actin (dilution 1:5000; Cat#66009-1-lg, Proteintech). Membranes were then incubated with the corresponding horseradish peroxidase-conjugated secondary antibodies (goat anti-rabbit IgG or anti-mouse IgG, Proteintech) at a 1:10,000 dilution for 1 h at room temperature. Immunoreactive bands were visualized using BeyoECL Moon (Cat# p0018FM, Beyotime) and quantified with ImageJ software (Version 1.52a, National Institutes of Health, Bethesda, MD, USA). Three biological replicates were performed.

### 2.6 Cell Viability Assay

Cell viability was assessed at 0, 24, 48, and 72 h intervals using a Cell Counting Kit-8 (CCK-8) assay kit (Cat# C0037, Beyotime Biotechnology). Briefly, cells were seeded in 96-well plates at a density of 5 × 10^3^ cells/100 μL per well. At each designated time point, 10 μL/well CCK-8 reagent was added to each well according to the manufacturer’s protocol. Following a 2 h incubation at 37°C, the absorbance values at 450 nm were measured using a microplate reader (BioTek Epoch, Winooski, VT, USA). Experimental design incorporated quintuplicate replicates per group with three independent biological repeats.

### 2.7 Cell Cycle Analysis

OVCAR-3 and A2780 cells were seeded in 6-well plates at a density of 6 × 10^5^ and 5 × 10^5^/mL per well, respectively, and cultured for 24 h at logarithmic growth phase. After trypsinizing, washing, and centrifuging, cells were fixed with 70% ethanol at −20°C for 4 h. Next, cells were resuspended with 500 μL PI/RNase Staining Buffer (Cat# 550825, BD Biosciences, San Jose, CA, USA) and incubated in the dark for 15 min. Subsequently, cell cycle distribution was analyzed by Beckman Coulter Flow Cytometry (Gallios System, Brea, CA, USA), collecting 15,000 events per sample, and evidence was processed using ModFit software (Version 5.0.9, Verity Software House, Topsham, ME, USA).

### 2.8 EdU Proliferation Assay

After seeding in 24-well plates at a density of 1 × 10^4^/well for 24 h, cells were labeled with BeyoClick™ EdU-555 (Cat# C0075S, Beyotime Biotechnology) based on the manufacturer’s protocols. After an additional 24 h incubation, images were acquired at 200× magnification using a BioTek Cytation C10 system (Agilent, Santa Clara, CA, USA). Proliferation rates were then quantified by calculating the red/blue fluorescence ratios with ImageJ software (Version 1.52a). Three biological replicates were performed.

### 2.9 Cell Migration, Invasion, and Wound Healing Assays

Transwell migration and invasion assays were conducted using a 6-well plate (Corning Inc., Corning, NY, USA). For both assays, cells (1 × 10^5^) were suspended in 300 μL of serum-free medium (OVCAR-3: RPMI-1640, Cat# KGL1503-500; A2780: DMEM, Cat# KGL1211-500, both from KeyGen Biotech) and seeded in the upper chamber. The lower chamber was filled with 600 μL of medium plus 10% FBS as a chemoattractant. The key distinction was that for the invasion assay, the upper chamber was pre-coated with Matrigel (Cat# YC356234, Yuanchuang Biotechnology Co., Ltd., Shanghai, China) at 10 μL Matrigel/90 μL of the same serum-free medium as the migration assay and fixed for 4 h. After 24 h incubation, cells that had migrated or invaded through the membrane were fixed, stained with Giemsa (Cat# G5637, Sigma-Aldrich Trading Co., Ltd., Shanghai, China), and counted. Three biological replicates were performed.

For a wound healing assay, cells were seeded in 6-well plates at a cell density of approximately 70% (3 × 10^5^). Upon reaching 80%–90% confluence, a uniform scratch was created in the cell monolayer using a sterile pipette tip. Wound closure rates were calculated from images captured at 0, 24, and 48 h intervals. The migration rate was calculated using the formula as follows: Migration rate (%) = [(A_0_ − A_t_)/A_0_] × 100%, where A_0_ stands for the scratch area at 0 h and A_t_ stands for the scratch area at the measured time point (e.g., 24 or 48 h). Three biological replicates were performed.

### 2.10 Colony Formation Assay

Following a 14-day culture period in 6-well plates (seeded at 1 × 10^4^/well), the colonies were fixed with 4% paraformaldehyde (PFA; Cat# JRY-31047, Jirongyuan Biotechnology Co., Ltd., Shanghai, China) for 30 min, stained with Crystal Violet (Cat# C6158-50G, Sigma-Aldrich Trading Co., Ltd.) for another 30 min. The colony was quantified using ImageJ software (Version 1.52a). Three biological replicates were performed.

### 2.11 Apoptosis Assay by Flow Cytometry

Apoptosis was detected using an FITC (fluorescein isothiocyanate)-Annexin V/propidium iodide (PI) double-staining assay. OVCAR-3 and A2780 cells were seeded in 6-well plates at a density of 6 × 10^5^/mL/well and 5 × 10^5^/mL/well, respectively, and cultured for 24 h. After washing with PBS, the cells were resuspended in 500 μL of 1× binding buffer. Subsequently, 3 μL FITC-Annexin V (Cat#556547, BD Biosciences, San Jose, CA, USA) and 5 μL PI (Cat#550825, BD Biosciences) were added to suspensions, followed by 15 min incubation in the dark. The population of apoptotic cells was measured using flow cytometry (Gallios System, Beckman Coulter), and the data were analyzed using FlowJo software (Version X.0.7, FlowJo, LLC, Ashland, OR, USA). Three biological replicates were performed.

### 2.12 Detection of Mitochondrial Membrane Potential (MMP), Reactive Oxygen Species (ROS), Lipid Peroxides, and Ferrous Iron (Fe^2+^)

For MMP detection, OVCAR-3 and A2780 cells were seeded in 6-well plates at a density of 6 × 10^5^ and 5 × 10^5^/well, respectively. Cells at 80% confluence were treated with JC-1 (JC-1 MitoMP Detection Kit, Cat# MT09, Dojindo, Kumamoto, Japan) (10 μg/mL in PBS) and incubated at 37°C for 30 min under light-protected conditions. After three washes with PBS, MMP in cells was evaluated by flow cytometric analysis (10,000 events/sample). For ROS detection, cells were incubated with DCFH-DA (ROS Assay Kit, Photo-oxidation Resistant DCFH-DA, R253, Dojindo) (10 μM) at 37°C for 30 min under light-protected conditions. The intracellular ROS levels were then measured by confocal microscopy after the free probes were removed. For lipid peroxide detection, cells were stained with 5 μM BODIPY-C11 (Liperfluo, L248, Dojindo) at 37°C for 30 min under light-protected conditions, followed by three PBS washes before flow cytometric analysis (10,000 events/sample). For Fe^2+^ detection, cells were treated with FerroOrange (FerroOrange, F374, Dojindo) (1 μM) and incubated in a 37°C/5% CO_2_ atmosphere for 30 min. After cells were washed thrice with HBSS, the intracellular Fe^2+^ level was assessed by confocal microscopy. Three biological replicates were performed. All procedures were performed according to the manufacturer’s instructions.

### 2.13 Treatment of Ferroptosis Inhibitor

Cells were plated in 96-well plates at a density of 5 × 10^3^ cells/well and incubated overnight at 37°C. The cells were then treated with the ferroptosis inhibitor Ferrostatin-1 (10 μM; Cat# SML0583-5MG, Sigma-Aldrich Trading Co., Ltd.) for 24 and 48 h. After the respective treatment periods, 10 μL of CCK-8 reagent was added to each well. Following an additional 2 h of incubation, the absorbance was measured at 450 nm using a microplate reader (SuPerMax 2800MF, Shanghai Shanpu Biotechnology, Shanghai, China). The cell viability was determined using the formula: Cell viability (%) = [(Absorbance of treated wells − Average absorbance of blank wells)/(Average absorbance of untreated control wells − Average absorbance of blank wells)] × 100%. Three biological replicates were performed.

### 2.14 Xenograft Mouse Model

Approval of animal studies was obtained from the Laboratory Animal Welfare and Ethics Committee of the Shanghai Public Health Clinical Center (Approval # 2022-A040-01) and was conducted in accordance with the ARRIVE guidelines (Animals in Research: Reporting *In Vivo* Experiments) [21]. To establish a xenograft model, 12 female BALB/c nude mice (5 weeks old and body weight range between 18–20 g; Shanghai Super-B&K, Shanghai, China) were housed in a specific pathogen-free class condition under 12 h light/dark cycles with controlled temperature (22 ± 1°C) and humidity (55%–60%). The mice were randomly assigned to two groups (n = 6 mice/group). The animals received a subcutaneous injection of 5 × 10^6^ sh-NC-infected OVCAR-3 cells (negative control group) or sh-KRT7-AS-infected OVCAR-3 cells (shRNA knockdown group) suspended in 100 μL serum-free medium. The predefined experimental endpoints were: (1) Dynamic tumor volume (monitored to reflect tumor growth kinetics) and (2) tumor weight (measured after euthanasia to reflect final tumor burden). Both endpoints were used as the key outcomes for statistical comparisons between the two groups in accordance with the institutional guidelines: (1) Maximum allowable tumor volume: ≤2000 mm^3^; (2) Body weight loss threshold: ≤15% of the initial body weight within a week; (3) Clinical signs of severe distress: persistent lethargy, loss of appetite, difficulty moving, abnormal posture (e.g., hunched back), or rough and unkempt fur. Tumor volume was monitored and recorded on days 1, 3, 5, 7, and 9 post-injection using the previously mentioned method [22]. No animals were removed from the study prior to the scheduled endpoint, as none of the above humane endpoint criteria were met during the entire experimental period. Mice were euthanized following anesthesia with 1% pentobarbital sodium (80 mg/kg, i.p.). A strict single-blind method was adopted during outcome assessment: the researchers responsible for tumor volume measurement, body weight recording, and general condition observation were unaware of the group allocation of the mice.

### 2.15 Immunohistochemical (IHC) and Hematoxylin-Eosin (H&E) Staining

Paraffin-embedded tissue sections (4 μm) were sequentially processed through deparaffinization and antigen retrieval by heating the sections in a 100°C EDTA buffer (Cat# PR40020, Proteintech) for 12 min. For IHC staining, permeabilization and blocking were performed at room temperature for 1 h using the Rabbit Two-step Detection Kit (Cat# PV-9001; ZSGB-BIO, Beijing, China), followed by incubation with primary antibody anti-PCNA (1:500; 24036-1-AP, Proteintech) at 4°C overnight. Subsequently, secondary antibody (1:500; PV-9001, ZSGB-BIO) was incubated for 1 h. After slides were washed 3 times with PBS (5 min each), freshly prepared Diaminobenzidine (DAB; Cat# PR30010, Proteintech) chromogenic detection was conducted for 5 min before hematoxylin counterstaining, dehydration, and mounting. For HE staining, deparaffinized sections were stained with hematoxylin (50 s), differentiated in 1% hydrochloric acid-ethanol (30 s), immersed in eosin (2 min), dehydrated, and mounted for brightfield microscopy. All procedures were strictly followed manufacturers’ protocols with three technical replicates per sample (n = 3). Three biological replicates were performed.

### 2.16 Statistical Analysis

Data were analyzed using IBM SPSS Statistics (Version 25.0; IBM SPSS Inc., Armonk, NY, USA), visualized with GraphPad Prism (Version 9.0.0, GraphPad Software Inc., San Diego, CA, USA), and presented as mean ± SD. Parametric tests (Student’s *t*-test for two-group comparisons; one-way ANOVA with Tukey’s post hoc for multi-group analyses) were applied to datasets with normal distribution and homogeneity of variance. To analyze prognostic correlation with lncRNAs between high and low-expression groups, a Kaplan-Meier survival plot with a median cutoff value was applied. Survival analyses utilized Kaplan-Meier curves with log-rank testing and Cox proportional hazards regression for hazard ratios (HR). For the subsequent validation of KRT7-AS, we evaluated its prognostic performance by constructing time-dependent Receiver Operating Characteristic (ROC) curves and calculating the corresponding Area Under the Curve (AUC). Significance threshold was *p* < 0.05.

## 3 Results

### 3.1 KRT7-AS Is Overexpressed and a Poor-Prognostic Marker in Ovarian Cancer

**Figure 1:**
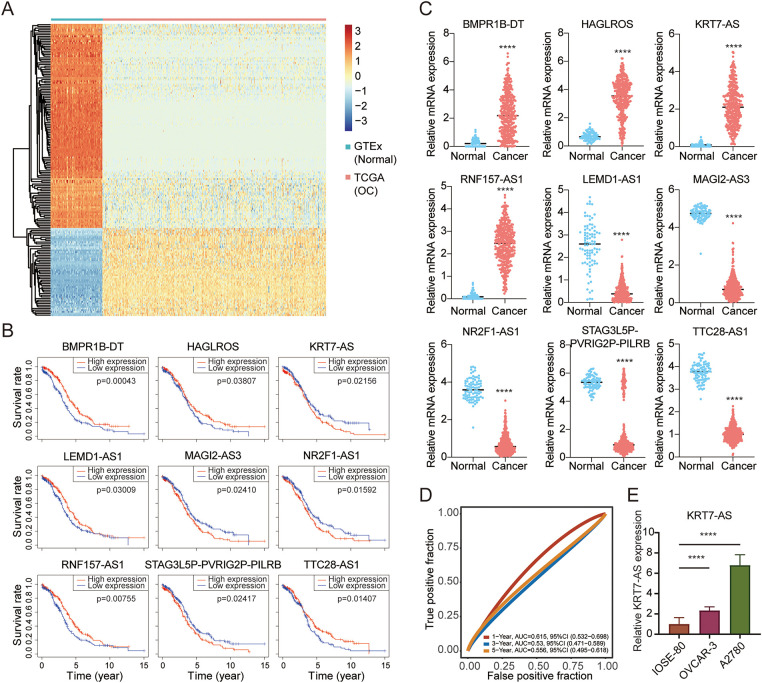
Characteristics of KRT7-AS expression associated with the prognosis of ovarian cancer patients. (**A**) Heatmap analysis of GTEx and TCGA datasets identified differential lncRNA expression (|Fold Change| > 2, *p* < 0.001) between normal ovarian tissues (Normal) and ovarian cancer tissues (OC) using log_2_-transformed normalized counts by log_2_(TPM + 1) for the color scale. Orange denotes upregulation and blue denotes downregulation. (**B**) Correlation analysis of survival rate with 9 lncRNAs between high and low expression groups by Kaplan-Meier plotter. The 177 differentially expressed lncRNAs were analyzed using the log-rank test. *p* < 0.05 was considered significant. (**C**) Expression validation of 9 candidate lncRNAs from RNA-sequencing expression profiles. Data in normal ovarian tissues (normal, n = 88) and ovarian cancer tissues (cancer, n = 374) were from the GTEx and TCGA databases, respectively. (**D**) The receiver operating characteristic (ROC) curves for the survival of patients with the elevated KRT7-AS by year 1, 3, and 5. The hazard ratio with 95% confidence intervals (95% CI) and log-rank *p*-value were calculated. The higher values of area under the curve (AUC) corresponded to higher predictive power. (**E**) KRT7-AS expression in normal ovarian surface epithelial cells (IOSE-80) and ovarian cancer cells (OVCAR-3 and A2780) by RT-qPCR. The unpaired Student’s *t*-test was used for a two-group comparison. ****p < 0.0001.

Utilizing the GTEx and TCGA databases to analyze lncRNA expression patterns from 88 normal ovarian tissues and 374 ovarian cancer tissues, we found 177 differentially expressed lncRNAs between normal and cancer tissues (|Fold Change| > 2, *p* < 0.001) (Fig. 1A). Among these lncRNAs, 52 were upregulated and 125 were downregulated in ovarian cancer. Further analysis of prognostic correlation with those lncRNAs between high and low-expression groups by a Kaplan-Meier survival plot with a median cutoff value identified 9 statistically significant candidates (BMPR1B-DT, HAGLROS, KRT7-AS, LEMD1-AS1, MAGI2-AS3, NR2F1-AS1, RNF157-AS1, STAG3L5P-PVRIG2P-PILRB, TTC28-AS1) (*p* < 0.05) using the log-rank test (Fig. 1B). RNA-sequencing expression profiles showed 4 upregulated lncRNAs (BMPR1B-DT, HAGLROS, KRT7-AS, RNF157-AS1) and 5 downregulated lncRNAs (LEMD1-AS1, MAGI2-AS3, NR2F1-AS1, STAG3L5P-PVRIG2P-PILRB, TTC28-AS1) in ovarian cancer tissues compared to normal tissues (Fig. 1C). Cross-validation of prognostic significance and expression profiles confirmed consistency in 3 lncRNAs that upregulated KRT7-AS had a poor prognosis, whereas downregulated LEMD1-AS1 and TTC28-AS1 had a favorable prognosis. Subsequent analysis of upregulated KRT7-AS as a prognostic marker using area under the curve (AUC) scores showed that the receiver operating characteristic (ROC) curves for survival by year 1, 3, and 5 were 0.615, 0.53, and 0.556, respectively (Fig. 1D). RT-qPCR confirmed that the level of KRT7-AS expression was higher in OVCAR-3 and A2780 cells than in IOSE-80 cells (Fig. 1E).

### 3.2 Knockdown of KRT7-AS Inhibits Ovarian Cancer Cell Proliferation

**Figure 2:**
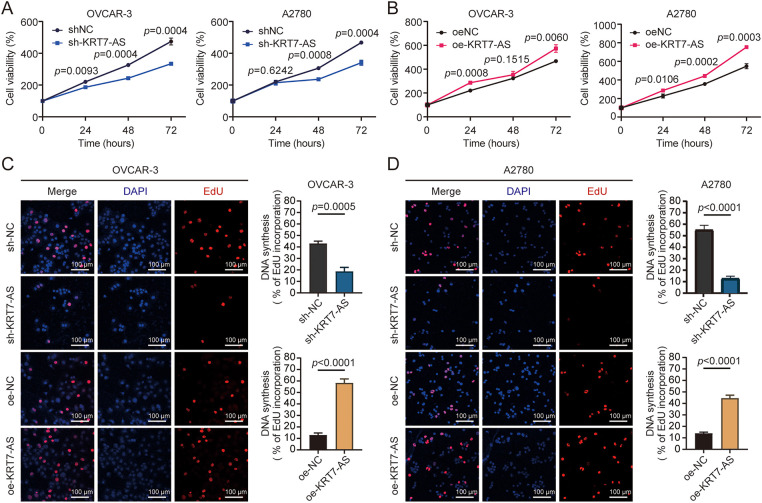

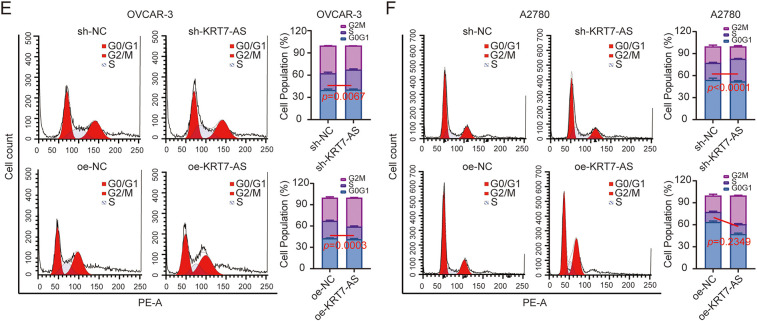
Effect of KRT7-AS on ovarian cancer cell proliferation. (**A**) Measurement of cell viability in OVCAR-3 and A2780 cells after sh-KRT7-AS infection by Cell Counting Kit-8 (CCK-8). (**B**) Measurement of cell viability in OVCAR-3 and A2780 cells after oe-KRT7-AS transfection by CCK-8. (**C**) Detection of cell proliferation by the EdU assay in OVCAR-3 cells after sh-KRT7-AS infection or oe-KRT7-AS transfection. (**D**) Detection of cell proliferation by the EdU assay in A2780 cells after sh-KRT7-AS infection or oe-KRT7-AS transfection. Signals in the EdU assay were detected using ImageJ software. Scale bar, 100 μm. (**E**) Detection of cell cycle by flow cytometry in OVCAR-3 cells after KRT7-AS knockdown or overexpression. (**F**) Detection of cell cycle by flow cytometry in A2780 cells after KRT7-AS knockdown or overexpression. Data were presented as mean ± SD (n = 3). The unpaired Student’s *t*-test was used for a two-group comparison. sh-NC, negative control shRNA; sh-KRT7-AS, KRT7-AS shRNA; oe-NC, negative control vector; oe-KRT7-AS, KRT7-AS overexpressing plasmid.

**Figure A1:**
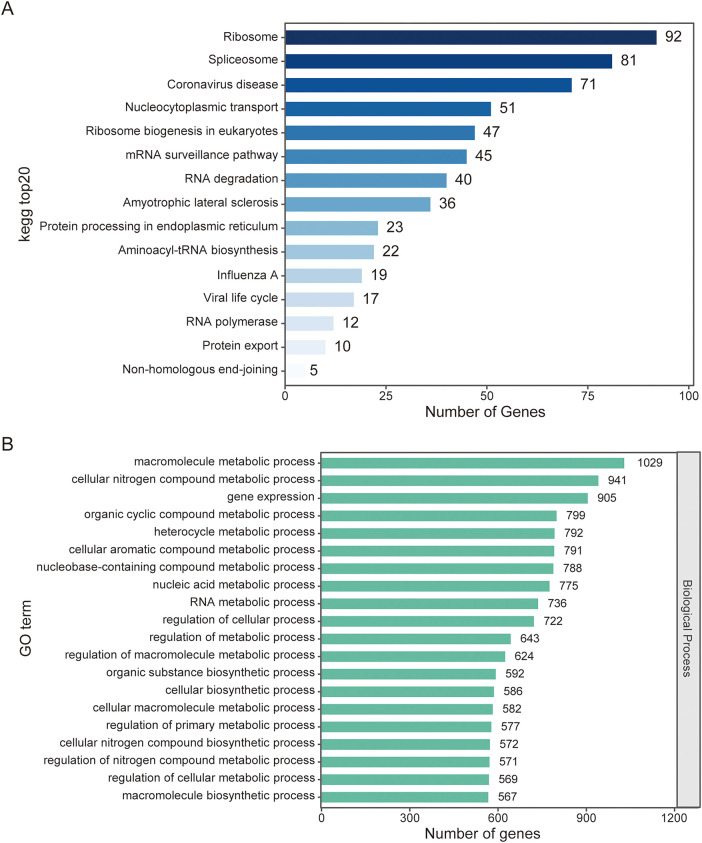
Pathways of candidate proteins that bind to KRT7-AS. (**A**) KEGG analysis of the top 20 pathways based on candidate proteins. The number in the square displays a gene ratio of each pathway on the x-axis. The color gradient represents the ratio size. (**B**) GO term analysis of the top 20 biological processes based on candidate proteins.

**Figure A2:**
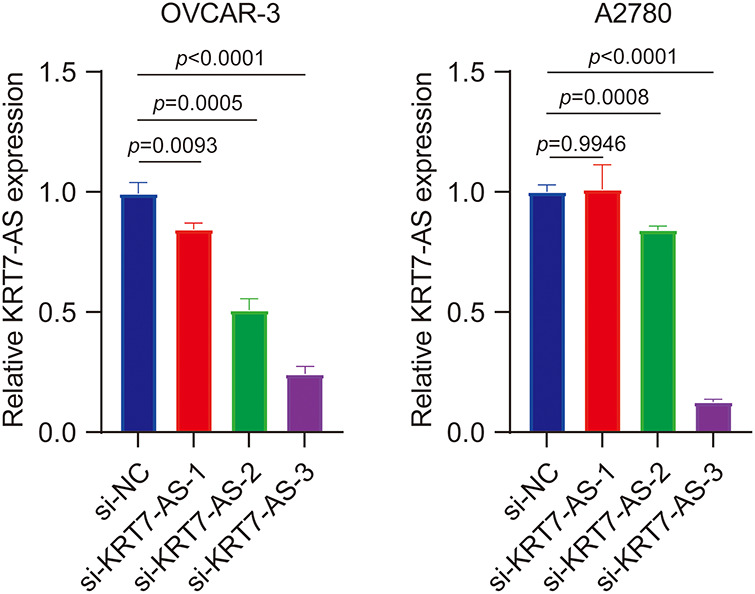
Detection of the knockdown efficacy of si-KRT7-AS by RT-qPCR in OVCAR-3 and A2780 cells after 3 siRNAs transfection. Data were presented as mean ± SD (n = 3). The unpaired Student’s *t*-test was used for a two-group comparison. si-NC, negative control siRNA; si-KRT7-AS, KRT7-AS siRNA.

**Figure A3:**
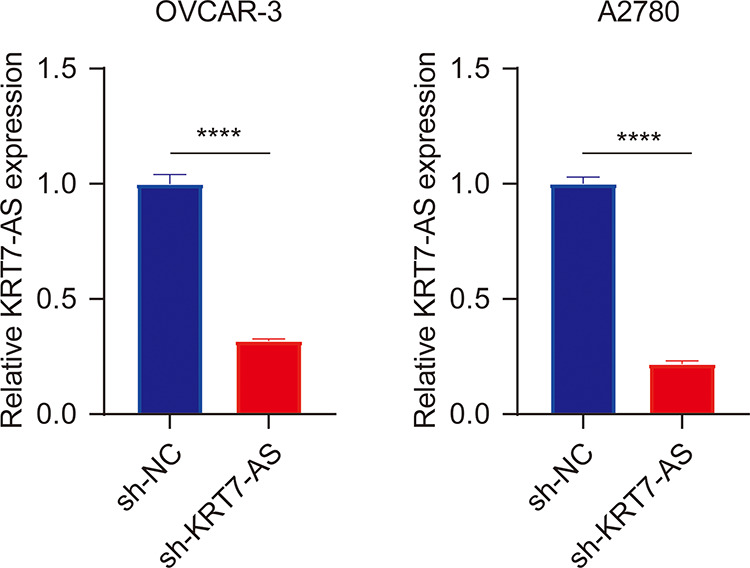
Detection of the knockdown efficacy of sh-KRT7-AS by RT-qPCR in OVCAR-3 and A2780 cells after shRNA infection. Data were presented as mean ± SD (n = 3). The unpaired Student’s *t*-test was used for a two-group comparison. ****p < 0.0001; sh-NC, negative control shRNA; sh-KRT7-AS, KRT7-AS shRNA.

**Figure A4:**
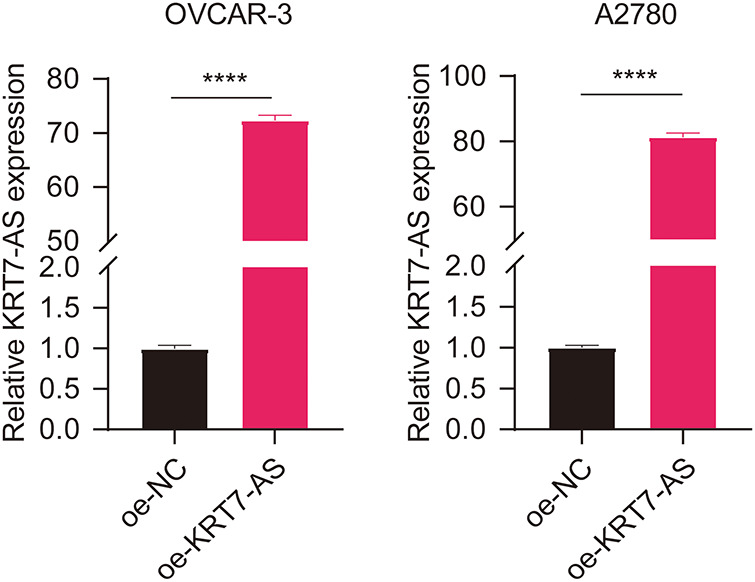
Detection of the overexpressing efficacy of oe-KRT7-AS by RT-qPCR in OVCAR-3 and A22789 cells after KRT7-AS overexpressing plasmid transfection. Data were presented as mean ± SD (n = 3). The unpaired Student’s *t*-test was used for a two-group comparison. ****p < 0.0001; oe-NC, negative control vector; oe-KRT7-AS, KRT7-AS overexpressing plasmid.

To predict the biological function of KRT7-AS, the bioinformatics analysis was applied using a biological database (http://service.tartaglialab.com/page/catrapid_omics2_group). Over a thousand proteins were predicted to contain RNA-binding domains (RBDs) that may potentially interact with KRT7-AS. Further analysis using KEGG (https://www.genome.jp/kegg/) and GO term (https://www.geneontology.org/) tools showed the top 20 pathways, including ribosome biogenesis, RNA degradation, and the regulation of cellular processes (Fig. A1A,B). To elucidate the function of KRT7-AS in ovarian cancer cells, the loss-of-function and gain-of-function approaches were applied. First, the knockdown efficacy of KRT7-AS was evaluated in OVCAR-3 and A2780 cells transfected with 3 siRNAs of KRT7-AS (Fig. A2). We found that si-KRT7-AS-3 was more efficient in ovarian cancer cells. Thus, the same sequence as si-KRT7-AS-3 was used to generate KRT7-AS-specific shRNA (sh-KRT7-AS). RT-qPCR confirmed the knockdown of KRT7-AS in OVCAR-3 and A2780 cells after lentivirus-packaged sh-KRT7-AS infection (Fig. A3). Conversely, the overexpression of KRT7-AS was also confirmed by RT-qPCR in ovarian cancer cells transfected with oe-KRT7-AS (Fig. A4). Subsequently, the KRT7-AS shRNA and the KRT7-AS overexpressing plasmid were used in a series of experiments. Compared to the negative control (NC), sh-KRT7-AS exhibited a decrease in OVCAR-3 and A2780 cell viability at 24, 48, and 72 h (Fig. 2A), whereas oe-KRT7-AS showed enhanced proliferative capacity (Fig. 2B). Furthermore, the EdU assay indicated that DNA replication was reduced in sh-KRT7-AS-infected ovarian cancer cells and DNA synthesis was increased in oe-KRT7-AS-transfected cells (Fig. 2C,D). Flow cytometry analysis revealed that knockdown of KRT7-AS induced the cell cycle arrest at the S phase, whereas overexpression of KRT7-AS reduced the duration of the S phase and facilitated the transition to the G2/M phase in OVCAR-3 and A2780 cells (Fig. 2E,F). These findings suggest that KRT7-AS-mediated regulation of S phase dynamics accelerates G2/M progression, thereby promoting ovarian cancer cell proliferation.

### 3.3 KRT7-AS Impacts on the Malignant Properties of Ovarian Cancer Cells

**Figure 3:**
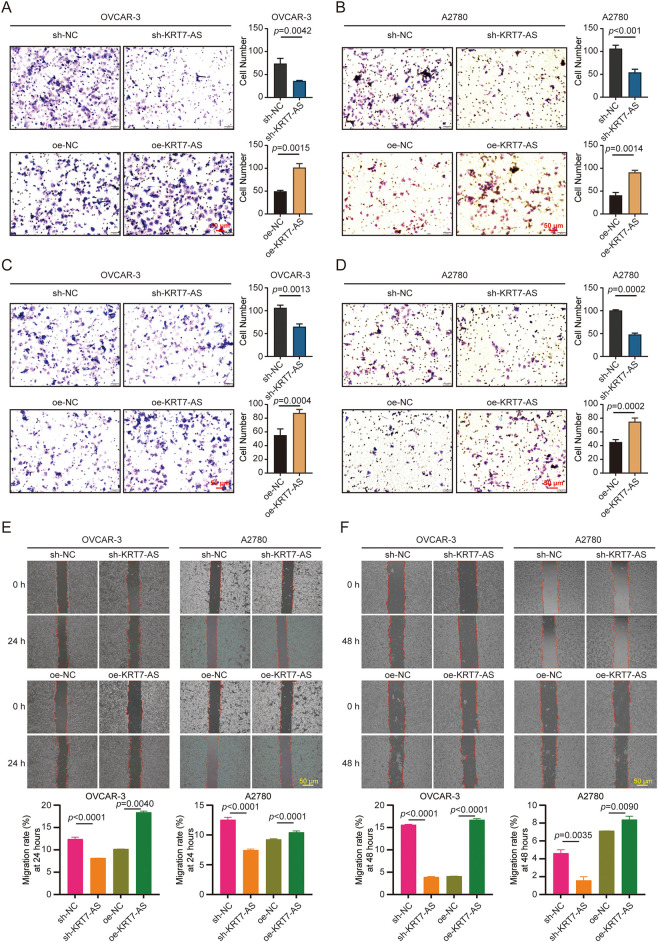
Effect of KRT7-AS on ovarian cancer cell migration, invasion, and wound healing. (**A**) Measurement of migration in OVCAR-3 cells after KRT7-AS knockdown or overexpression. (**B**) Measurement of migration in A2780 cells after KRT7-AS knockdown or overexpression. (**C**) Measurement of invasion in OVCAR-3 cells after KRT7-AS knockdown or overexpression. (**D**) Measurement of invasion in A2780 cells after KRT7-AS knockdown or overexpression. Scale bar, 50 μm. (**E**) Measurement of wound healing in OVCAR-3 and A2780 cells after KRT7-AS knockdown or overexpression at 24 h. (**F**) Measurement of wound healing in OVCAR-3 and A2780 cells after KRT7-AS knockdown or overexpression at 48 h. Migration rate (%) was calculated. Scale bar, 50 μm. Data were presented as mean ± SD (n = 3). The unpaired Student’s *t*-test was used for a two-group comparison. sh-NC, negative control shRNA; sh-KRT7-AS, KRT7-AS shRNA; oe-NC, negative control vector; oe-KRT7-AS, KRT7-AS overexpressing plasmid.

Next, transwell assays demonstrated that KRT7-AS knockdown significantly reduced the migration and invasion capacities of OVCAR-3 and A2780 cells, whereas its overexpression enhanced these malignant properties (Fig. 3A–D). Scratch wound healing assays further revealed that silencing KRT7-AS markedly impaired cellular migration capacity, as evidenced by reduced wound closure compared to controls, whereas its overexpression accelerated migration velocity (Fig. 3E,F). These outcomes substantiate the regulatory impact of KRT7-AS on ovarian cancer cell motility.

### 3.4 Regulation of Apoptosis by KRT7-AS Is Inconsistent in Ovarian Cancer Cells

**Figure A5:**
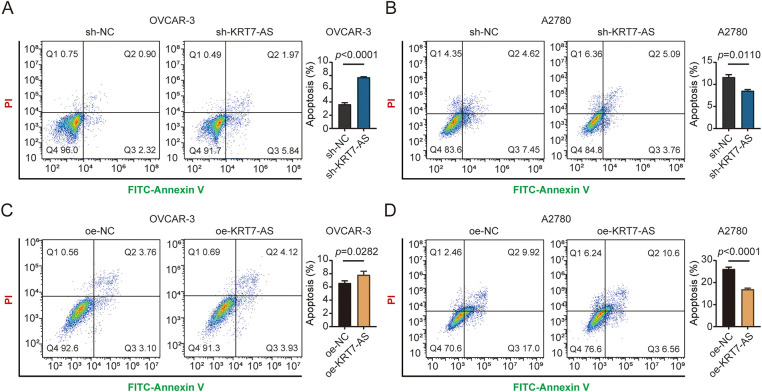
Detection of apoptotic cells in ovarian cancer cells by flow cytometry. (**A**) Detection of apoptotic cells after KRT7-AS knockdown in OVCAR-3 cells. (**B**) Detection of apoptotic cells after KRT7-AS knockdown in A2780 cells. (**C**) Detection of apoptotic cells after KRT7-AS overexpression in OVCAR-3 cells. (**D**) Detection of apoptotic cells after KRT7-AS overexpression in A2780 cells. Histograms show the number of apoptotic cells as a percentage. Data were presented as mean ± SD (n = 3). The unpaired Student’s *t*-test was used for a two-group comparison. FITC, fluorescein isothiocyanate; PI, propidium iodide; sh-NC, negative control shRNA; sh-KRT7-AS, KRT7-AS shRNA; oe-NC, negative control vector; oe-KRT7-AS, KRT7-AS overexpressing plasmid.

**Figure A6:**
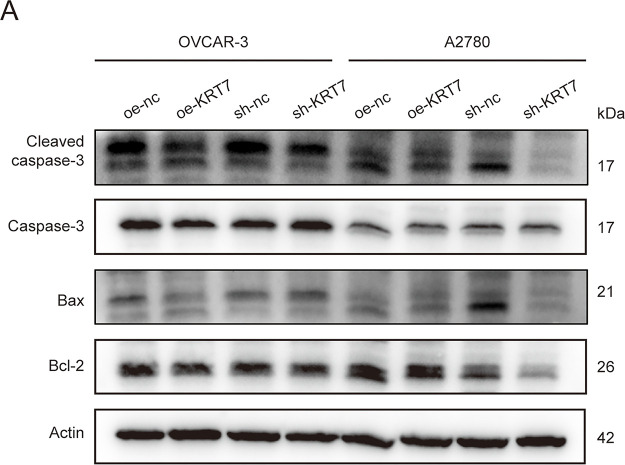

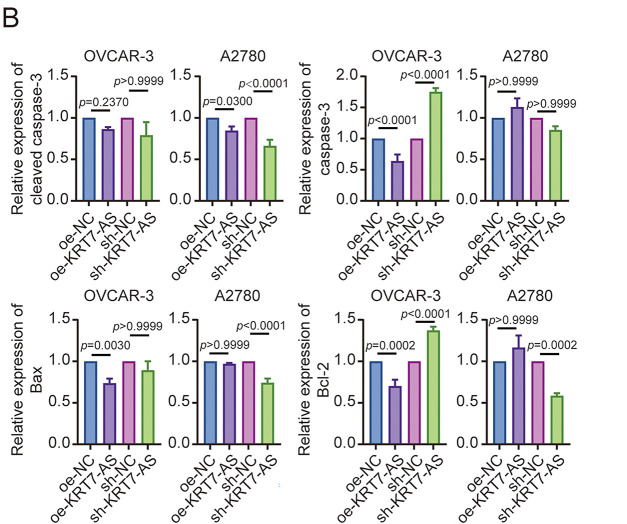
Detection of apoptosis-related proteins by Western blot in ovarian cancer cells. (**A**) Detection of cleaved caspase 3, total Caspase 3, Bax, Bcl-2, and Actin in OVCAR-3 and A2780 cells after KRT7-AS overexpression or knockdown. Representative blots are shown. (**B**) Histograms show semi-quantification of protein bands from Western blotting gels in (**A**). Data were presented as mean ± SD (n = 3). The unpaired Student’s *t*-test was used for a two-group comparison. sh-NC, negative control shRNA; sh-KRT7-AS, KRT7-AS shRNA; oe-NC, negative control vector; oe-KRT7-AS, KRT7-AS overexpressing plasmid.

Interestingly, apoptosis assessment indicated variable effects of KRT7-AS knockdown and overexpression in terms of the regulation of apoptosis rates in OVCAR-3 and A2780 cells detected by flow cytometry (Fig. A5A–D). Next, apoptosis-related proteins such as cleaved caspase-3, total caspase-3, Bax, and Bcl-2 were detected by Western blot in two ovarian cancer cell lines (Fig. A6A). Neither overexpression nor knockdown of KRT7-AS affected cleaved caspase-3 expression in OVCAR-3 cells and caspase-3 in A2780 cells (Fig. A6B). All experiments were repeated at least 3 times. Due to the discrepant outcomes between the two cell lines, further investigation to explore alternative regulatory mechanisms underlying KRT7-AS-mediated cell death, such as ferroptosis, was pursued.

### 3.5 Knockdown of KRT7-AS Suppresses Ovarian Cancer Progression by Promoting Ferroptosis

**Figure 4:**
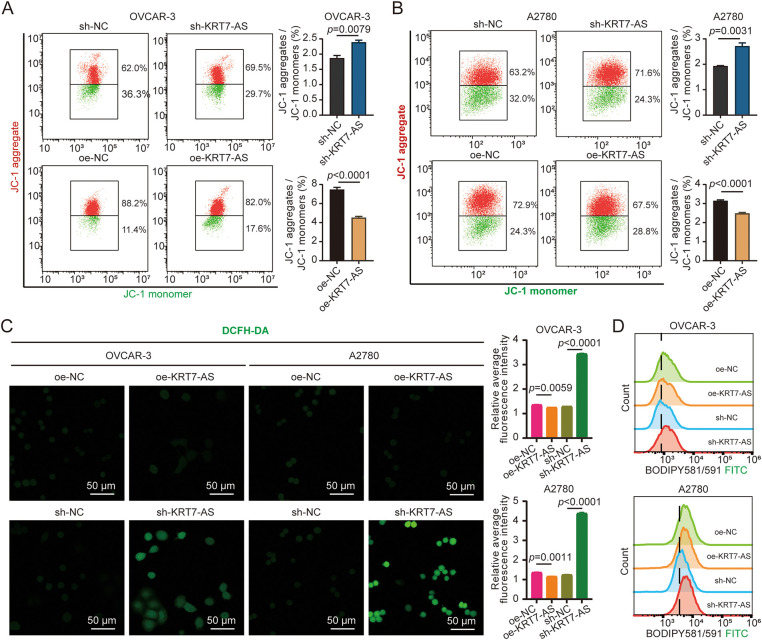
Measurement of mitochondrial membrane potential (MMP), intracellular reactive oxygen species (ROS), and lipid peroxidation in ovarian cancer cells after KRT7-AS knockdown or overexpression. (**A**) Detection of MMP (JC-1) by flow cytometry in OVCAR-3 cells after KRT7-AS knockdown or overexpression. (**B**) Detection of JC-1 by flow cytometry in A2780 cells after KRT7-AS knockdown or overexpression. (**C**) Detection of ROS (DCFH-DA) in OVCAR-3 and A2780 cells after KRT7-AS knockdown or overexpression by fluorescence confocal microscopy. Scale bar, 50 μm. The relative DCFH-DA intensity was quantified (right panel). (**D**) Detection of lipid peroxidation (BODIPY) in OVCAR-3 and A2780 cells after KRT7-AS knockdown or overexpression by fluorescence confocal microscopy and quantified by FlowJo. Data were presented as mean ± SD (n = 3). The unpaired Student’s *t*-test was used for a two-group comparison. FITC, fluorescein isothiocyanate; sh-NC, negative control shRNA; sh-KRT7-AS, KRT7-AS shRNA; oe-NC, negative control vector; oe-KRT7-AS, KRT7-AS overexpressing plasmid.

To elucidate the mechanism by which KRT7-AS mediates cell death, first of all, we assessed MMP. KRT7-AS knockdown increased the MMP levels, whereas its overexpression decreased the MMP levels, in both OVCAR-3 and A2780 cells (Fig. 4A,B). In light of an elevation in MMP as a consequence of ferroptosis, next, we proceeded to measure intracellular ROS, lipid peroxidation, and ferrous iron (Fe^2+^) to substantiate the role of KRT7-AS in driving ferroptosis. Notably, silencing KRT7-AS significantly increased ROS levels (Fig. 4C), whereas overexpression of KRT7-AS had the opposite effect. Subsequently, we found that knockdown of KRT7-AS enhanced lipid peroxidation (Fig. 4D), in which lipid peroxide was stained with 5.0 μM BODIPY-C11 and detected by flow cytometric analysis.

**Figure 5:**
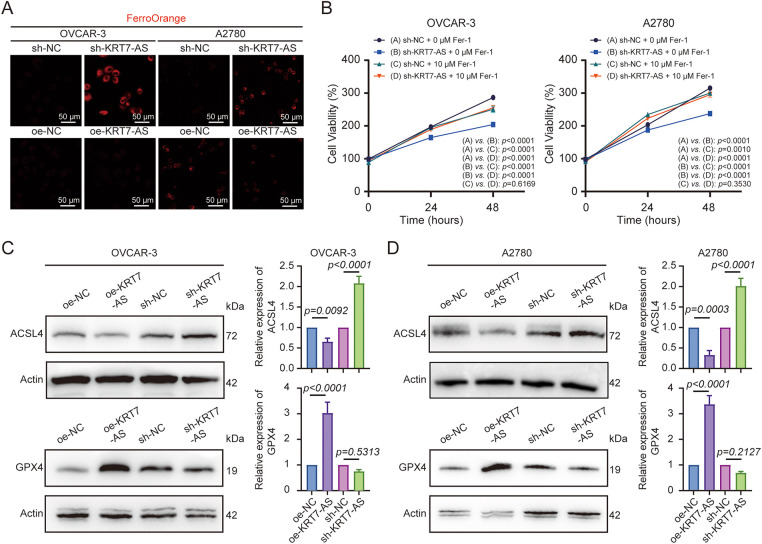
Effect of KRT7-AS on ferroptosis in ovarian cancer cells. (**A**) Detection of intracellular Fe^2+^ (FerroOrange) in OVCAR-3 and A2780 cells after KRT7-AS knockdown or overexpression by fluorescence confocal microscopy. Scale bar, 50 μm. (**B**) Rescue experiments confirmed the cell viability induced by KRT7-AS knockdown in the presence of the ferroptosis inhibitor Ferrostatin-1 (Fer-1). (**C**,**D**) Detection of ferroptosis-related proteins ACSL4 and GPX4 by Western blot in OVCAR-3 and A2780 cells after KRT7-AS overexpression or knockdown. A representative blot is shown. Histograms show semi-quantification of protein bands from gels. Data were presented as mean ± SD (n = 3). The one-way ANOVA followed by Tukey’s test was used for multiple group comparison (**B**). The unpaired Student’s *t*-test was used for a two-group comparison (**C**,**D**). sh-NC, negative control shRNA; sh-KRT7-AS, KRT7-AS shRNA; oe-NC, negative control vector; oe-KRT7-AS, KRT7-AS overexpressing plasmid.

Remarkably, silencing KRT7-AS specifically induced the accumulation of Fe^2+^ (Fig. 5A), whereas overexpression of KRT7-AS reduced Fe^2+^. Rescue experiments confirmed that the reduction in cell viability induced by KRT7-AS knockdown was rescued in the presence of a ferroptosis inhibitor Ferrostatin-1 (Fig. 5B). Furthermore, ferroptosis-related proteins ACSL4 and GPX4 were further evaluated by Western blot analysis (Fig. 5C,D). Overexpression of KRT7-AS decreased ACSL4 protein levels, whereas knockdown of KRT7-AS increased ACSL4 protein levels, in both OVCAR-3 and A2780 cells. GPX4 protein was increased in KRT7-AS-overexpressing OVCAR-3 and A2780 cells but not in KRT7-AS-knockdown cells. Collectively, these findings indicate that the depletion of KRT7-AS can activate the ferroptotic cascade in ovarian cancer cells, further indicating the role of KRT7-AS in the ferroptosis pathway.

### 3.6 Knockdown of KRT7-AS Suppresses Ovarian Cancer Progression

**Figure 6:**
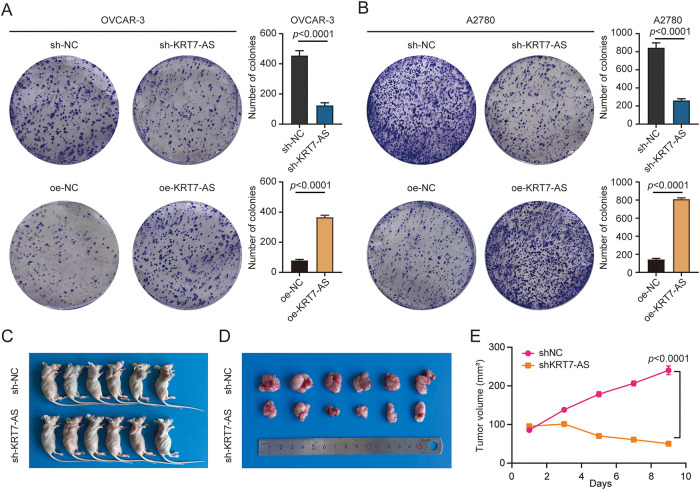

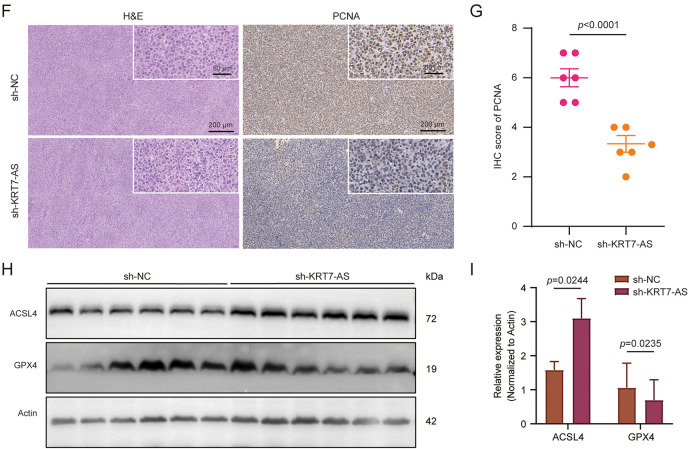
Effect of KRT7-AS on tumor formation. (**A**) Cell colony formation in OVCAR-3 cells after KRT7-AS knockdown or overexpression. (**B**) Cell colony formation in A2780 cells after KRT7-AS knockdown or overexpression. Data were presented as mean ± SD (n = 3). (**C**) The xenograft mouse model was generated by subcutaneous injection with sh-KRT7-AS-infected OVCAR-3 cells. (**D**) Tumors were isolated and photographed after the animals were sacrificed. (**E**) Measurement of tumor volume in sh-NC and sh-KRT7-AS groups. (**F**) Detection of PCNA proteins in the tumor samples of the xenograft mice by IHC staining. Representative images are shown. Original scale bar, 200 μm (50 μm in small image). (**G**) Statistical analysis of (**F**) using ImageJ software. (**H**) Detection of ferroptosis-related proteins ACSL4 and GPX4 in the tumor samples of the xenograft mice by Western blot. (**I**) Semi-quantification of the gels from (**H**). Animal data were presented as mean ± SD (n = 6). The unpaired Student’s *t*-test was used for a two-group comparison. sh-NC, negative control shRNA; sh-KRT7-AS, KRT7-AS shRNA; oe-NC, negative control vector; oe-KRT7-AS, KRT7-AS overexpressing plasmid.

KRT7-AS knockdown significantly reduced, whereas its overexpression significantly enhanced, colony-forming ability in OVCAR-3 and A2780 cells (Fig. 6A,B). *In vivo* studies further demonstrated that knockdown of KRT7-AS significantly inhibits tumor growth in xenograft mouse models, as evidenced by decreased tumor dimensions and volumes in the sh-KRT7-AS group compared to the control (Fig. 6C–E). Subsequently, IHC evaluation showed that KRT7-AS knockdown significantly decreased proliferating marker PCNA protein in sh-KRT7-AS tissues compared to sh-NC (Fig. 6F,G). Furthermore, Western blot analysis of sh-KRT7-AS tumor tissues showed the upregulation of ACSL4 and downregulation of GPX4 protein expression (Fig. 6H,I). Taken together, these data suggest that silencing KRT7-AS exerts tumor-suppressive effects.

## 4 Discussion

The current study identifies KRT7-AS as an oncogenic lncRNA in ovarian cancer. Overexpressed KRT7-AS is a marker of poor prognosis in patients with ovarian cancer and has an impact on the malignant properties in ovarian cancer cells. Functionally, knockdown of KRT7-AS inhibits ovarian cancer cell proliferation, migration, and invasion, and suppresses ovarian cancer progression by activating ferroptosis.

KRT7-AS is an antisense transcript of keratin 7 known to be involved in the immune microenvironment, cell proliferation, and metastasis in multiple cancers [19,20,23]. It has been shown that the dysregulation of lncRNAs has been identified to mediate diverse biological functions in ovarian cancer [24,25]. In breast cancer, KRT7-AS stability is enhanced through METTL3-mediated m6A modification, facilitating cancer progression [26]. Furthermore, KRT7-AS forms RNA-RNA hybrids with KRT7 to regulate its expression, thereby promoting gastric cancer proliferation and metastasis [19]. A recent bioinformatics analysis revealed immunomodulatory potential of KRT7-AS and its inclusion in prognostic lncRNA models for ovarian cancer [27]. Moreover, the established associations between m7G-associated KRT7-AS and ovarian carcinogenesis suggest that prognostic models incorporating these molecules may provide clinical utility in guiding prognostic evaluation, assessing immunotherapy responses, and screening chemotherapeutic agents for patients with ovarian cancer [28]. While the biological role of KRT7-AS in ovarian carcinogenesis remains to be elucidated, existing evidence from KRT7-related pathways and its oncogenic functions in other malignancies provides a compelling rationale for investigating its potential involvement in ovarian cancer metastasis, though mechanistic exploration requires systematic investigation.

Through bioinformatics analysis with cell-based approaches, this study provides comprehensive evidence that KRT7-AS acts as an oncogene in ovarian cancer. KRT7-AS was highly expressed in ovarian cancer tissues and correlated with poor prognosis. Functional experiments revealed that KRT7-AS overexpression promoted cell proliferation, migration, and invasion, while its knockdown suppressed tumor growth. Targeting KRT7-AS may represent a promising therapeutic strategy for patients with ovarian cancer, which was further validated by *in vivo* experiments, demonstrating tumor growth inhibition upon KRT7-AS downregulation. These oncogenic effects were primarily governed through the suppression of ferroptosis, driven by KRT7-AS-mediated downregulation of ACSL4 and upregulation of GPX4, ultimately facilitating tumor progression. The OVCAR-3 cell line is derived from a patient with high-grade serous ovarian adenocarcinoma. In contrast, the A2780 cell line is derived from a patient with ovarian endometrioid adenocarcinoma. Based on sequence, epigenomic, and expression analyses, OVCAR-3 cells exhibit overexpression of *CCNE1* and *AKT2*, whereas A2780 cells exhibit underexpression of *CDKN2A* and *TGFBR2* [29], indicating the existence of differences between these two cell lines. Thus, the inconsistent apoptosis results in OVCAR-3 and A2780 cells were most likely attributed to the heterogeneity of the cell lines. Because cell death after KRT7-AS knockdown was observed in OVCAR-3 and A2780 cells, we pivoted our subsequent research on ferroptosis rather than apoptosis.

Ferroptosis is one type of programmed cell death, characterized distinctly by its reliance on iron and its oxidative mechanism of cellular destruction [11], facilitating distinct genetic, biochemical, morphological, and metabolic characteristics compared to other cell death types [30]. Notably, ferroptosis propagates rapidly in wave-like patterns across cell populations. Morphologically, ferroptotic cells exhibit mitochondrial irregularities, including outer membrane swelling, changes in cristae density, and their breakdown. Dysregulated lipid peroxidation driven by iron and Fenton-like reactions breaks down lipid membranes, a feature of ferroptosis. Ultimately, the execution of ferroptosis is regulated by interconnected pathways involving iron, glutathione (GSH), and lipid metabolism [31]. This form of cell death is triggered by iron-dependent peroxidation of phospholipids containing polyunsaturated fatty acids (PUFAs) [32–34]. Excess iron stored in ferritin is released and degraded upon cellular stimulation, leading to massive Fe^3+^ release. This Fe^3+^ is converted to Fe^2+^ via transferrin receptor-1 (TFR1) and ultimately deposited into cytoplasmic iron pools, thereby increasing ROS levels and causing deleterious effects [35–37]. As ferroptosis is an iron-dependent, lipid-peroxidation-driven form of non-apoptotic cell death [38,39], the key hallmarks include the accumulation of iron, increased lipid peroxidation, transcriptional upregulation of ACSL4, and inactivation of GPX4, which collectively lead to oxidative membrane damage. These established features form the basis of current detection strategies, which typically combine biochemical assays with gene expression profiling. The current experimental approaches were effective for detecting ferroptosis in cell and tissue contexts. Utilizing a suite of cellular and biochemical assays to assess hallmarks of ferroptosis, we consistently demonstrate that KRT7-AS knockdown triggers a ferroptotic phenotype: it increased ACSL4 protein levels, decreased GPX4 protein levels, enhanced iron and lipid peroxidation accumulation, and elevated MMP and ROS levels. Critically, the suppression of cell viability induced by sh-KRT7-AS was rescued by the ferroptosis inhibitor Ferrostatin-1. These data clearly indicate the occurrence of ferroptosis in OVCAR-3 and A2780 cells following KRT7-AS knockdown.

It has been demonstrated that ferroptosis-related proteins such as ACSL4 and GPX4 play the pivotal roles, which function as positive and negative regulators of ferroptosis, respectively. ACSL4 serves as an enzyme to trigger ferroptosis, specifically acting to promote the process [40]. GPX4 prevents ferroptosis by decreasing cellular ROS levels and repairing lipid oxidation-induced damage [41]. However, GPX4 deactivation due to GSH reduction or direct suppression by RSL3 or FIN56 disrupts antioxidant capacity, resulting in excessive lipid ROS production and initiating ferroptosis via dysregulated lipid peroxidation [42,43]. The current study revealed that KRT7-AS did not mediate tumor progression through apoptotic regulation, but demonstrated that KRT7-AS knockdown suppressed malignant phenotypes in ovarian cancer through the induction of ferroptosis. Emerging evidence indicates that specific lncRNAs can attenuate tumor malignancy by modulating ferroptosis-related pathways [44,45]. In light of these mechanistic insights, we speculate that KRT7-AS functionally interacts with ferroptosis pathways, necessitating focused investigation into their crosstalk to elucidate the biological basis of KRT7-AS-mediated oncogenesis. Subsequent investigation of ferroptosis-related phenotypes revealed that KRT7-AS knockdown significantly increased intracellular ROS levels and iron accumulation, consistent with its functional involvement in ferroptosis.

Some limitations of the study still exist. First, the current study lacks clinical validation. Future studies should include prospective data as well as retrospective data analyses to evaluate the role of KRT7-AS. Additional clinical cohorts and samples are required. Second, the biogenesis of KRT7-AS in ovarian cancer is not fully understood and requires further investigation. Some molecular mechanisms in greater depth regarding the upstream regulators and downstream targets of KRT7-AS should be followed. Third, whether KRT7-AS interacts with its potential molecules has not been proven and needs to be verified. Therefore, more accountable experiments would be worthwhile to pursue and require further research.

## 5 Conclusions

Elevated KRT7-AS expression correlates with poor prognosis of patients with ovarian cancer. Functional studies demonstrate its role in promoting cancer cell proliferation, invasion, metastasis, and colony formation. KRT7-AS knockdown leads to the activation of the ferroptotic cascade and the inhibition of tumor progression. Thus, targeting KRT7-AS may have clinical potential and therapeutic value.

## Data Availability

The data that support the findings of this study are available from the Corresponding authors upon reasonable request.

## Appendix A

**Table A1:** Sequences of siRNAs.

siRNA	Sequence (5^′^ to 3^′^)
si-KRT7-AS-1	
Sense	GGGACUGUUAUCAUUUCAATT
Antisense	UUGAAAUGAUAACAGUCCCTT
si-KRT7-AS-2	
Sense	GCAGAAACAUGUCCUACGUTT
Antisense	ACGUAGGACAUGUUUCUGCTT
si-KRT7-AS-3	
Sense	GCUCUAGUGAUCUGUGUGATT
Antisense	UCACACAGAUCACUAGAGCTT
si-NC	
Sense	UUCUCCGAACGUGUCACGUTT
Antisense	ACGUGACACGUUCGGAGAATT

Note: si-KRT7-AS-1, first siRNA of KRT7-AS; si-KRT7-AS-2, second siRNA of KRT7-AS; si-KRT7-AS-3, third siRNA of KRT7-AS; si-NC, scramble sequence of negative control.

**Table A2:** Oligonucleotide sequence of shRNA-KRT7-AS.

	Sequence (5^′^ to 3^′^)
Sense	GATCCGCTCTAGTGATCTGTGTGATTCAAGAGATCACACAGATC ACTAGAGCTTTTTTG
Antisense	AATTCAAAAAAGCTCTAGTGATCTGTGTGATCTCTTGAA TCACACAGATCACTAGAGCG

**Table A3:** Primer sequences of oe-KRT7-AS.

Primer	Sequence (5^′^ to 3^′^)
Forward	CTACCGGACTCAGATCTCGAGGGAGCCTCGCTGGCCTCTGCG
Reverse	GTACCGTCGACTGCAGAATTCAATGATCACAATAGCATTTATTAAG

Note: oe-KRT7-AS, overexpressing plasmid of KRT7-AS.

**Table A4:** Primers for RT-qPCR.

Primer	Sequence (5^′^ to 3^′^)
KRT7-AS	
Forward	TCCAACGCCTATGTTCCAGTTC
Reverse	ACATTGTGCCACGGACATCTTG
Actin	
Forward	TCATCACCATTGGCAATGAG
Reverse	CACTGTGTTGGCGTACAGGT
